# Impact of long working hours on health based on observations in China

**DOI:** 10.1186/s12889-021-11190-0

**Published:** 2021-07-07

**Authors:** Liming Chu

**Affiliations:** School of Labor Economics, Capital University of Economics and Business, Beijing, 100070 China

**Keywords:** Long working hours, Self-rated health, CMP, China

## Abstract

**Background:**

Health should be a key focus in considerations of long working hours. Little is known about for which groups of people working longer hours is more harmful to their health. Additionally, the definition of long working hours varies slightly due to country differences in working hours systems. Therefore, this study aims to explore the association between long working hours and the self-rated health (SRH) level, taking into account gender and educational differences.

**Method:**

Data were collected from two waves (2016 and 2018) of the China Family Panel Studies (CFPS). A total of 6972 workers were available for analysis. Descriptive statistical analysis, an ordered probit (oprobit) model and conditional mixed process (CMP) regression analyses were used to analyze the data. Furthermore, I conducted a stratified analysis by gender and education groups.

**Result:**

This study observed a negative association between long working hours and SRH. Compared to other education groups, labor with long working hours had a more negative impact on the SRH of those with higher education. Long working hours had a more negative influence on the SRH of male workers. In contrast, no clear association was found among female workers.

**Conclusion:**

This study estimates SRH of those with long working hours in China. Among workers, long working hours have a negative impact on the health of workers with college degrees or beyond. One possible explanation is that they do not exercise, their diet is unreasonable, and their working conditions involve chronic exposure to computer radiation. The negative health effects of long working hours on males are four times greater than those on females. This study provides valuable insights into the health of the workforce, working time regulations and overtime rules.

## Background

In the era of fast economic development, the quality of the labor force has drawn widespread attention, and in this regard, health is an important factor. In recent years, excessive labor has slowly become common due to the continuously extended working hours of workers, which have a direct impact on their health level and harms economic development and social stability. A better scholarly understanding of this topic appears to be urgently needed. China published the Health China 2030 plan, which aims to provide every citizen with health services by 2030. China’s basic medical insurance system has reached the goal of covering both urban and rural residents, meeting their medical needs, and providing a strong guarantee for the improvement of workforce health. Understanding the impact of labor market performance on health holds great significance for exploring the influencing factors in the health level of the labor force.

Workforce health can be measured in two ways. The first is the self-assessment of health status, which can more intuitively reflect the health status of individuals, show a closer relationship with studies on individual choices such as labor force participation and retirement, and acquire data more easily [1–3]. The second is the construction of comprehensive indicators. Regarding activities of daily living (ADLs), little variation in young populations is shown [4]. The Short Form 36 (SF-36) health survey questionnaire [5], Euroqol-5D [6], Health Utilities Index (HUI) [7], and Quality of Well-Being Scale (QWB) [8] which includes objective indicators based on individual health status, are also limited. Reflecting an individual’s subjective assessment of his or her health status, quality of life indicators are very rigorous in their data requirements. Subjective measures, such as self-assessed health (SAH) or satisfaction with one’s own health, provide valuable insights because they might respond more quickly to changes in working time than objective indicators, such as the frequency of doctor visits or absences from work due to sickness.

Since the 1980s, working hours have been a matter of debate and have become a focal issue in Canada, France, Germany, the United States, the United Kingdom, and other Western countries [9, 10]. As a universal phenomenon among most organizations and companies, longer working hours are motivated by the possibility of job advancement or better employment opportunities and higher wages. According to efficiency wage theory, the cost of unemployment is positively correlated with the number of working hours, whose increase is an important indication of work effort but can be harmful to the health of the population above a certain level, with some degree of overtime being associated with high levels of stress and health effects accumulating over time [11–15].

In general, related studies emphasize that health suffers from underemployment and, in particular, unemployment [16–20]. In addition, an extensive literature documents that long working hours are closely related to chronic diseases, poor mental health and unhealthy behaviors such as smoking and alcohol consumption [21–25]. Epidemiological research has shown that long working hours affect the risk of cardiovascular diseases [26–29], stress, chronic fatigue [30], the probability of stroke [31], anxiety, sleep quality, all-cause mortality, smoking, hypertension, self-perceived health, health behaviors and mental health status [32, 33]. Regarding long working hours, similar results have been obtained by other studies, including myocardial infarction [34], injuries, poor physical health, alcohol consumption [35], physical inactivity [36], and depression [37]. In general, it has been found that long working hours are detrimental to health [38]. While studies conclude that additional working hours have adverse consequences for health, they predominantly focus on specific symptoms.

Mild differences between occupations in labor and heterogeneity in time commitment can have different effects on health, with more resources and energy invested in career development leading to more distinct occupational identities and highly complex jobs producing more significant effects on health [39]. Gender differences also exist in the effects of long working hours on health. Mainly due to the differences in the social roles and socioeconomic status of different gender groups, involuntary overwork is positively associated with the poor mental health of males but can lead to the poor physical health of females [40]. Long working hours can be detrimental to both individual and spousal health.

Despite the significance of long working hours, a meta-analysis conducted by Sparks and Cooper [25] discovered that a variety of health syndromes are slightly positively correlated with long working hours based on a relevant literature review. The research perspectives were mostly analyzed based on one aspect, such as the relationship between long working hours and the health status of a certain group of migrant workers, while heterogeneity analysis focused on special groups and occupational types. No subsequent research on the association between long working hours and health or combined medical insurance has been conducted. The issue of long working hours is quite important in every society, which thus highlights the need to investigate the connection between long working hours and the health of workers. Moreover, the link between long working hours and health is rarely studied from the perspective of different genders or educational levels.

Given the insufficient understanding of some details of this issue, this study investigated the association between long working hours and self-rated health (SRH) using data from the China Family Panel Studies (CFPS). This study aimed to examine (1) the definition of long working hours and (2) whether long working hours are associated with SRH, with particular attention to differences in gender and education.

## Data and methods

### Data

Involving 25 provinces, municipalities, and autonomous regions, the data adopted in this paper were sourced from the CFPS and four main questionnaires, namely, community, family, adult, and child questionnaires, which can be used for research on individual microbehaviors. In this paper, the 2016 and 2018 data were combined for a panel data analysis based on the nonfarm labor force aged 18–65 as subjects. A total of 6972 valid samples were obtained after the deletion of missing, extreme, and unreasonable values.

### Measurement of the variables

#### Measurement of the health level of the labor force

In this study, the outcome variable was the SRH level. The question “What do you consider your health to be?” was chosen to measure the health level of the labor force based on the design of the CFPS questionnaire. The response options were “unhealthy”, “fair”, “relatively healthy”, “healthy” and “very healthy”, which were assigned a value of 1, 2, 3, 4 and 5, respectively. As shown in Table 1, the average SRH level of the respondents was fair.

**Table 1:** Definition of variables and descriptive statistics

Variables	Definition	Full sample (*N* = 6972)		Working hours are less than 50 h (*N* = 3419)		Working hours not less than 50 h(*N* = 3553)	
		Mean	Standard Error	Mean	Standard Error	Mean	Standard Error
Self-rated health	Unhealthy = 1,fair = 2,relatively healthy =3, healthy = 4, very healthy = 5	3.21	1.05	3.23	1.01	3.19	1.10
Long work hours	Whether working time is less than 50 h	0.51	0.5	—	—	—	—
Gender	Male = 1,female = 0	0.29	0.46	0.26	0.44	0.33	0.47
Age	age	36.74	11.06	36.38	11.03	37.08	11.09
Income	Annual personal income	8.42	4.03	9.06	3.55	7.80	4.36
Level of education	Junior secondary and below = 1, senior secondary/technical school/vocational high school = 2, junior college and above = 3	1.84	0.88	2.15	0.89	1.54	0.77
Marital status	At the wedding =1,otherwise = 0	0.80	0.40	0.77	0.42	0.82	0.39
Whether to smoke or not	Smoking = 1,otherwise = 0	0.05	0.22	0.04	0.19	0.06	0.24
Whether to drink or not	Three times a week in the past month = 1	0.07	0.25	0.06	0.24	0.08	0.27
Exercise Frequency	How many times a week do you do exercise	2.16	2.92	2.40	2.93	1.93	2.89
Whether you have health insurance or not	Health Insurance = 1, No = 0	0.90	0.30	0.91	0.29	0.89	0.31

#### Measurement of long working hours

In this paper, the core explanatory variable was long working hours. Based on the design of the CFPS questionnaire, the question “How many hours a week does this job typically involve?” was chosen to measure working hours. Referring to the related Chinese and international research and according to the working time restrictions in the Labor Law of China, this paper used a working time of more than 50 working hours a week as the standard for identifying long working hours. Articles 36, 38, and 41 of the Labor Code stipulate that a worker should not work more than 8 h a day and 44 h a week on average. Employers should ensure at least 1 day of rest per week for workers. Employers may extend working hours, which should generally not exceed 1 hur per day for production and business purposes, as suggested by the worker and trade union; additionally, if there is a need to extend work hours for special reasons, employers should not extend working hours more than 3 h per day and more than 36 h per month to guarantee the physical health of workers. As a result, the reference standard working time is 44 h, and the maximum weekly working time can be 44 + 1 * 6 = 50 h in view of production and operational needs. Therefore, in this paper, a working time of more than 50 h a week for workers was regarded as overwork, which is consistent with the standard of measuring overwork in China and internationally. As illustrated in Table 1, the labor force works 52 h a week, exceeding the working hours standard.

#### Measurements of the covariates

To reduce the underlying bias in the statistical model caused by omitted variables, the empirical analysis controlled for variables including gender, age, educational level, personal income, marital status, smoking, drinking, exercise frequency, and medical insurance participation, which are specifically defined in Table 1.

### Ordered Probit model

This paper focused on the relationship between long working hours and the physical health of the labor force, and the explanatory variable, SRH, as multi-ordered data, was unsuitable for ordinary least squares (OLS) estimation. As an extension of the probit model dealing specifically with cases in which the interpreted variable is sorted data, the following model was set:

$$ {\mathrm{health}}_{i,t}=F\left(\beta {longworkinghours}_{i,t}+\gamma {X}_{i,t}+{\varepsilon}_{i,t}\right) $$

where health_*i*, *t*_ represents the dependent variable; *β* refers to the intercept term; *X*_*i*, *t*_ means a series of control variables; *γ* represents for the coefficient of the influence of these control variables on the health level of the respondents; *ε*_*i*, *t*_ is a random error term; and F (∙) denotes a nonlinear function that takes the following form:

$$ \mathrm{F}\left({y}_{i,t}^{\ast}\right)=\left\{\begin{array}{cc}1& {y}_{i,t}^{\ast}\le {C}_1\\ {}2& {C}_1<{y}_{i,t}^{\ast}\le {C}_2\\ {}\mathrm{M}& \mathrm{M}\\ {}J& {y}_{i,t}^{\ast }>{C}_{j-1}\end{array}\right. $$

where ${y}_{i,t}^{\ast }$ represents the latent variable, satisfying

$$ {y}_{i,t}^{\ast }=\beta {\mathrm{workhour}}_{i,t}+\gamma {X}_{i,t}+{\varepsilon}_{i,t} $$

C_1_ < C_2_ < … < C_J-1_ are tangent points and parameters to be estimated.

$$ \mathrm{P}\left({\mathrm{y}}_{i,t}=1|{X}_{i,t}\right)=P\left({y}_{i,t}^{\ast }<{C}_1\right) $$

$$ \mathrm{P}\left({\mathrm{y}}_{i,t}=2|{X}_{i,t}\right)=P\left({C}_1\le {y}_{i,t}^{\ast }<{C}_2\right) $$

$$ \mathrm{P}\left({\mathrm{y}}_{i,t}=3|{X}_{i,t}\right)=P\left({C}_2\le {y}_{i,t}^{\ast }<{C}_3\right) $$

$$ \mathrm{P}\left({\mathrm{y}}_{i,t}=J|{X}_{i,t}\right)=1-P\left({C}_{n-2}\le {y}_{i,t}^{\ast }<{C}_{n-1}\right) $$

If the random error term is in the normal distribution, the probability of a higher health level of the labor force will increase.

A causal relationship may exist between long working hours and other variables such as health. For example, a person will find it difficult to work if he or she is not in a healthy state. To solve the endogeneity of long working hours and to identify the econometric model, whether the average working time of industry is less than 50 h was used as the instrumental variable of working time. Whether the working time in the same trade is too long will affect the working time of individuals but have no impact on the health of individuals. However, in this paper, health is a discrete variable, and two-stage least squares based on a continuous variable may fail [41]. Therefore, conditional mixed process (CMP) regression analyses and extended regression models (ERMs) that can handle endogeneity were used to re-estimate the baseline regression model.

## Results

The regression results of the relationship between long working hours and the health level of individuals are reported in Table 2. Columns (1)–(3) are the ordered probit (oprobit), ERM, and CMP regressions results, which suggest that, all other things being equal, long working hours were negatively correlated with health across the entire sample; these results were significant at the 5–10% levels. An increase in working hours will diminish returns and lead to a decline in the SRH of the workforce.

**Table 2:** Relationship between long working hours and individual health level

	OPROBIT	ERM	CMP
Long working hours	−0.0519^*^	− 0.0729^*^	− 0.0681^**^
	(0.090)	(0.050)	(0.047)
Gender	0.150^***^	0.151^***^	0.139^***^
	(0.000)	(0.000)	(0.000)
Age	−0.0275^***^	−0.0274^***^	− 0.0254^***^
	(0.000)	(0.000)	(0.000)
Income	0.00772^**^	0.00764^**^	0.00718^**^
	(0.035)	(0.045)	(0.039)
Level of education	−0.0182	−0.0180	−0.0163
	(0.334)	(0.324)	(0.334)
Marital status	0.0519	0.0516	0.0481
	(0.182)	(0.176)	(0.169)
Smoking	−0.138^**^	−0.138^**^	−0.129^**^
	(0.043)	(0.039)	(0.035)
Drinking	0.133^**^	0.132^**^	0.123^**^
	(0.022)	(0.026)	(0.024)
Exercise	0.0264^***^	0.0263^***^	0.0246^***^
	(0.000)	(0.000)	(0.000)
Medical insurance	−0.0662	−0.0297	−0.0276
	(0.168)	(0.607)	(0.606)
Year	YES	YES	YES
Province	YES	YES	YES
sigma2_u	0.168		
_cons	(0.310)		
n		0.834^***^	0.834^***^
		(0.000)	(0.000)
_cons		0.106^***^	
		(0.000)	
Log likelihood			−10,170.721
Wald		135.97***	
Observations	6972	6972	6972

Notes: *p* value in parentheses; * *p* < 0.1, ** *p* < 0.05 and *** *p* < 0.01.

The OLS and endogeneity processing model results show that long working hours are harmful to health.

The analysis above shows that the health level of the labor force is constrained by long working hours, but individuals’ behavior is generally dominated by their beliefs, value orientation and attitudes towards realistic interests. Different cultures form different ideas and lifestyles, which ultimately exerts a direct or indirect impact on people’s health. Thus, the impact of long working hours on the health level of the labor force varies by gender and education.

Table 3 divides the sample by educational level and gender and further reports the relationship between long working hours and the health level of the labor force among different samples grouped by gender and educational level. From the perspective of differences in educational level, long working hours have a more significant impact on the SRH of workers with a college degree or above and a limited impact on the SRH of workers with other levels of educational attainment. From the perspective of gender differences, long working hours have a more significant influence on the SRH of male workers, with this result being significant at the 1% level.

**Table 3:** Relationship between long working hours and the self-rated health of workforce among different age stages and education levels

	Below junior college	Tertiary and above	Male	Female
Long working hours	0.0167	− 0.294^***^	−0.156^**^	− 0.0394
	(0.709)	(0.000)	(0.022)	(0.325)
Gender	0.158^***^	0.132^**^	—	—
	(0.000)	(0.013)	—	—
Age	−0.0236^***^	− 0.0329^***^	− 0.0223^***^	− 0.0273^***^
	(0.000)	(0.000)	(0.000)	(0.000)
Income	0.00865^**^	−0.00458	0.0126^**^	0.00459
	(0.019)	(0.578)	(0.047)	(0.272)
Level of education			−0.0347	−0.00783
			(0.265)	(0.698)
Marital status	0.0544	0.0789	0.0108	0.0522
	(0.252)	(0.175)	(0.864)	(0.221)
Smoking	−0.107	−0.245^*^	− 0.0980	− 0.361^**^
	(0.140)	(0.054)	(0.133)	(0.041)
Drinking	0.0893	0.235^**^	0.167^**^	0.0202
	(0.136)	(0.047)	(0.011)	(0.841)
Exercise Frequency	0.0164^***^	0.0564^***^	0.0312^***^	0.0222^***^
	(0.002)	(0.000)	(0.001)	(0.000)
Medical Insurance	−0.0925	0.0942	0.101	−0.0705
	(0.228)	(0.306)	(0.316)	(0.265)
Year	YES	YES	YES	YES
Province	YES	YES	YES	YES
Observations Observations	4722	2250	2053	4919
Log likelihood	− 6889.171	− 2976.8187	− 3117.2115	− 6990.6308

Notes: *p* value in parentheses; * *p* < 0.1, ** *p* < 0.05 and *** *p* < 0.01.

## Discussion

This paper focused on investigating the impact of long working hours on the health of the workforce using the CMP regression analyses. Based on the literature on the effects of workforce health levels, the variables were strictly controlled to address the estimation bias resulting from omitted variables and to ensure that the results were credible.

### The health of the workforce depends on the combination of long working hours, education, income levels and other individual characteristics

Across the entire sample, long working hours were negatively correlated with health, that finding is consistent with previous reports [3, 36]. An increase in working hours will diminish returns and lead to a decline in the SRH of the workforce. By participating in work activities, workers can give full play to their physical and social functions, but they can no longer obtain a sense of pleasure and belonging. Even if wage returns increase, the mental and physical strength of the workforce will be impaired. A gradual decline in the SRH level of the labor force will occur, increasing the risk factors for depression, hypertension, and other diseases [26, 42, 43].

### Participation in medical insurance has no significant effect on self-rated health

Across the entire sample, long working hours were negatively correlated with health, and participation in medical insurance failed to significantly improve the SRH level of the labor force In fact, it showed a downward trend. With a security model oriented towards “treatment, prevention, hospitalization and outpatient care”, China’s medical insurance system ignores disease prevention and outpatient treatment, and it fails to intervene in the workforce in a timely manner to eliminate the causes of workers’ physical discomfort. In China, the basic medical insurance for urban workers mainly covers inpatient medical expenses beyond the minimum payment line, and medical expenses exceeding the maximum payment line need to be paid by individuals. In some regions, medical expenses for general outpatient care are paid by individual or individual account of medical insurance. In order not to delay working hours or save expenses, the workforce physical appear “Minor illness pain” more choice to wait for disease self-cure or buy medicine self-treatment, coupled with the labor force to disease prevention awareness is not strong, health level can not be improved. The medical insurance system “focus on treatment, neglect prevention ”of the security model ignored in the physical examination, maintenance and promotion of health protection.

### The relationship between long working hours and self-rated health varies by education and gender

The study also demonstrates that long working hours have a more significant impact on the SRH of workers with higher levels of educational attainment. To some extent, the increase in the working hours of workers with higher educational levels is detrimental to their level of health, this is also supported by many other studies conducted in Korea [44], possibly because of the psychological pressure exerted by the self-expectations that they hold with regard to their occupations as well as the difference between giving and receiving. A difference can be found in the time allocation between rest and workdays. The key to the decline in SRH lies in the factors that influence the physical and psychological aspects of an individual, such as a lack of exercise, an unreasonable diet, and working conditions that involve chronic exposure to computer radiation. Workers with lower educational levels are mostly engaged in alternative work or repetitive manual labor, while those with lower skill levels are concentrated in low-end labor-intensive industries and services. Increased working hours have a limited impact on the health of these workers.

Long working hours had a significant impact on the health of the male labor force. In the distribution of family roles, males have traditionally been breadwinners, focusing more on their careers and being more susceptible to workplace stressors [45, 46]. Men have to endure more stress and work longer hours than women, while women are more likely to play multiple roles at work and at home. Longer working hours have a limited impact on the health of women.

## Conclusions

Using the nonfarm employed workforce and the 2016 and 2018 waves of the CFPS, this paper examined the relationship between long working hours and workforce health through the lens of working hours. It found that long working hours were negatively correlated with health and had a more significant impact on the SRH of workers with higher levels of educational attainment. The effects of long working hours on the SRH of the workforce vary by education and gender. This finding has great implications for future research, which should pay attention to regulating working time, carrying out a comprehensive health and hygiene education.

This study offers several implications for working time regulations and overtime rules to protect workers’ health. In the short term, it may seem that long working hours for employees may be beneficial for maximizing the profits of companies, and increased working hours may result in higher performance. However, health is an important human capital factor for workers, and long working hours cannot contribute to sustained productivity gains over a longer period. They lead to an excessive drain on the personal energy of workers, crowding out time that could be spent to invest in health and resulting in more work stress. This phenomenon can cause more work and mental stress and physical health problems, and it can even lead to the forced cessation of work. The analysis in this paper shows that the average working hours of the workforce exceed the requirements of the Labor Law of China, which thus makes it necessary to limit the excessive working hours of the workforce and promote the regularization of working hours in the labor market. Moreover, the regulations related to overtime hours should be further standardized, and employers should pay wages for the overtime hours of the workforce based on relevant legal standards to reduce the damage of excessive working hours to the health level of the workforce and to facilitate the balance between workers’ work and nonwork days.

Although it is consistent with the characteristics of most Western countries in the period of rapid economic development, excessive labor harms the interests of workers and sustainable economic development. While maintaining sustained and healthy economic development, on the one hand, the government should raise the knowledge levels and skills of workers through formal education and vocational training, targeting workers with lower skill levels to increase their productivity. On the other hand, higher education should be the key to solving the health problems associated with education. Carrying out health education in universities and changing life habits that are harmful to health provide directions for deepening the comprehensive hygiene education in China.

The empirical analysis shows that whether the labor force participates in health insurance has no significant impact on workers’ SRH. From the promulgation of the Opinions on the Overall Planning of the Basic Medical Insurance System for Urban and Rural Residents to the completion of the integration of the system in the vast majority of regions in China, medical insurance has been able to meet more medical needs and has expanded its coverage, basically achieving the goal of universal health insurance. However, some problems remain to be solved in the implementation of China’s basic medical insurance system, which was originally established with the intention that all individuals facing the risk of diseases should share the risk to resist the financial and health impacts brought by diseases. Compared with commercial medical insurance, basic medical insurance can solve the problem of adverse selection. However, China has set up a basic medical insurance system for different groups of people instead of implementing a single basic medical insurance system. In terms of the payment for and treatment provided by basic medical insurance, there exists a large gap between urban workers and urban and rural residents. If they are able to make a choice, some healthy young people will choose to participate in the basic medical insurance of urban and rural residents, which involves low payment levels. To reduce the resistance of system integration, many regions have adopted the policy of “Single System, Multi-Standard”. Based on the premium level chosen, insured individuals can enjoy the corresponding treatment provided by medical insurance [47, 48]. In practice, the “Single System, Multi-Standard” can easily cause an adverse selection of insured individuals, who will choose a high premium level if in poor health, which is not conducive to the long-term stable operation of health insurance funds. As a measure for promoting the integration of the basic medical insurance system, it is important to abolish the practice of “Single System, Multi-Standard”, increase the financial subsidies of low-income groups, ensure the full coverage of basic medical insurance, and truly unify the reimbursement provided by medical insurance.

## Data Availability

The datasets used during the current study are available from the website of the Institute of Social Science Survey (http://isss.pku.edu.cn/cfps/download/login) and can be accessed by registering to access the study data. The datasets analyzed during the current study are available from the corresponding author upon reasonable request.
